# High-Throughput BLI for One-Step Anti-*Pseudomonas plecoglossicida* IgM Detection in *Larimichthys crocea* Serum

**DOI:** 10.3390/ijms27093897

**Published:** 2026-04-27

**Authors:** Qiuye Shao, Yuan Wang, Junfang Zhou, Shiming Peng, Peng Wang, Xincang Li

**Affiliations:** 1 School of Health Sciences and Engineering, University of Shanghai for Science and Technology, Shanghai 200093, China; qiuye1102@163.com; 2 East China Sea Fishery Research Institute, Chinese Academy of Fishery Sciences, Shanghai 200090, China; wy9501@yeah.net (Y.W.); jfzhou126@126.com (J.Z.); shiming.peng@163.com (S.P.)

**Keywords:** immunoglobulin M, avidity and quantification, biolayer interferometry (BLI), *Larimichthys crocea*

## Abstract

Accurate measurement of antigen-specific antibody responses is essential for evaluating antibody avidity and quantification. Traditional. Enzyme-Linked Immunosorbent Assay (ELISA), while widely used, is limited by lengthy procedures, dependence on secondary antibodies, and inconsistent reproducibility. In this study, biolayer interferometry (BLI) was established and validated for simultaneous quantification and avidity assessment of specific IgM in serum of *Larimichthys crocea* (Large yellow croaker) using *Pseudomonas plecoglossicida* outer membrane protein Omp-H as antigen. Sera from immunized and control fish were analyzed by both BLI and ELISA, with systematic comparison between platforms. Optimal serum dilutions were 1:128 for BLI and 1:1024 for ELISA. Validation with another outer membrane protein, Omp-W, confirmed the method’s broad applicability. BLI association signals and avidity indices correlated strongly with ELISA values, yielding consistent results for both antigens. BLI successfully captured specific antibody responses in infected sera and demonstrated superior inter-plate reproducibility compared to ELISA, which exhibited significant inter-plate variation. However, BLI required lower serum dilutions (hence larger volumes) to achieve comparable sensitivity. These findings establish BLI as a rapid, single-step method providing reliable quantitative and avidity data for teleost IgM, offering a reproducible alternative to ELISA with potential applications in vaccine evaluation and aquaculture infection detection.

## 1. Introduction

The intensification of the aquaculture industry, while meeting the growing global demand for protein, has also rendered farmed aquatic organisms increasingly vulnerable to severe disease outbreaks [1]. *Larimichthys crocea* (Large yellow croaker), one of the most economically valuable and widely cultivated marine fish species along the southeastern coast of China, supports an industry whose health is directly tied to the livelihoods of fishermen and regional economic stability [2]. However, the frequent occurrence of major diseases, including bacterial, viral, and parasitic infections, has caused substantial economic losses to the *L. crocea* aquaculture industry [3]. Among these, *P. plecoglossicida* has emerged as a major pathogen causing visceral white spot disease, a highly destructive condition that severely impacts the aquaculture of *L. crocea* [4]. In this context, a thorough understanding of its immune defense mechanisms, coupled with the development of effective immune surveillance tools, is critical for early disease detection, vaccine evaluation, and health management of farmed populations [5]. Immunoglobulin M (IgM), the earliest evolved and predominant antibody isotype in fish during the primary immune response, serves as a key serological indicator of immune status [6]. Therefore, establishing an accurate, sensitive, efficient, and high-throughput method suitable for large-scale quantification of serum IgM in *L. crocea* is not only essential for fundamental research in fish immunology but also provides indispensable technical support for achieving precision aquaculture and effective disease control [7].

A mouse monoclonal antibody specific to the constant region of the *L. crocea* IgM heavy chain serves as an ideal core reagent for developing immunoassays [7]. In fish immunology, the enzyme-linked immunosorbent assay (ELISA) continues to be the primary method for serum antibody quantification, valued for its highly mature technology, streamlined protocols, adaptability to high-throughput processing, and economic efficiency [8]. However, traditional ELISA has inherent limitations that are becoming increasingly evident under the demands of modern high-throughput and high-precision research [9]. First, its procedure is laborious and multi-stepped, typically involving antigen/antibody coating, blocking, repeated incubation and washing cycles, enzymatic color development, and reaction termination, a process that is time-consuming, often requiring several hours to complete [10]. This not only demands considerable operator skill but also renders the assay susceptible to human error at multiple steps, compromising inter-assay reproducibility [11]. Furthermore, its sensitivity is constrained by the detection limits of enzymatic colorimetric methods (ng/mL), restricting its utility for analyzing trace samples with very low antibody concentrations [12]. Additionally, ELISA is susceptible to sample matrix effects, as complex serum components can adsorb non-specifically to the solid phase, interfering with specific antigen–antibody binding and resulting in elevated background noise or signal suppression [13].

Biolayer Interferometry (BLI), a rapidly evolving new-generation label-free biophysical technology, offers an attractive solution to the aforementioned problems [14]. The core principle of BLI involves the direct, real-time quantification of biomolecular interactions by monitoring the shift in the interference spectrum at the tip of a biosensor resulting from molecular association or dissociation [15]. By monitoring molecular interactions in real time, BLI generates a complete association-equilibrium-dissociation curve within minutes, from which the kinetic parameters, including the association rate constant (ka), dissociation rate constant (kd), and equilibrium dissociation constant (KD), can be directly calculated [16]. This provides a far more in-depth quantitative description of the molecular interaction mechanism, exceeding the analytical capacity of ELISA [17]. The technique is simple, rapid, and requires minimal sample volumes, a feature particularly advantageous for analyzing scarce serum from juvenile *L. crocea* or conducting multi-parameter analyses on precious samples [18]. Currently, BLI technology has proven to be a powerful tool in fields such as drug development, protein engineering, and antibody screening [14,19]. However, its application to the detection and quantitative analysis of immunoglobulins in aquatic animals, particularly fish, remains in its early stages. A systematic empirical investigation is still required to validate its performance advantages over traditional ELISA for detecting antibodies in *P. plecoglossicida* infection serum samples. Therefore, this study, leveraging the previously generated mouse monoclonal antibody, aims to conduct a comprehensive investigation spanning from method development to performance comparison [20]. This will provide objective data to inform technology selection in fish immunology. Here, we describe the pioneering establishment of a novel high-throughput, single-step BLI-based method for detecting IgM in *L. crocea* following *P. plecoglossicida* infection.

## 2. Results

### 2.1. Serum IgM Levels and Avidity Measured by ELISA

**Figure 1:**
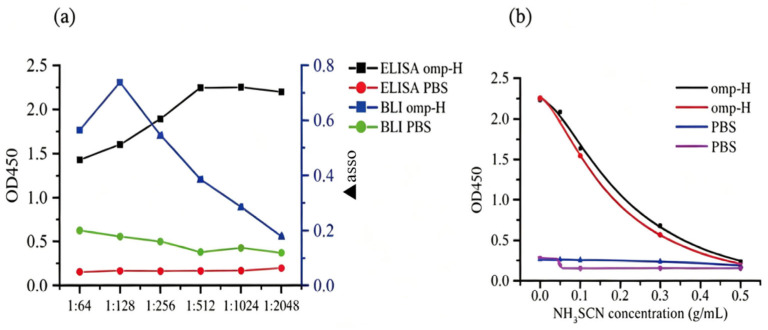
Dynamics of Serum Antibody Response and Its Correlation with Avidity Decay in Omp-H-Immunized *L. crocea*. (**a**) Correlation between serum ELISA absorbance at 450 nm (left axis) and BLI wavelength shift (▲asso, right axis) following immunization with Omp-H. (**b**) Analysis of the logarithmic decay model of *L. crocea* serum by ACE ELISA.

**Figure 2:**
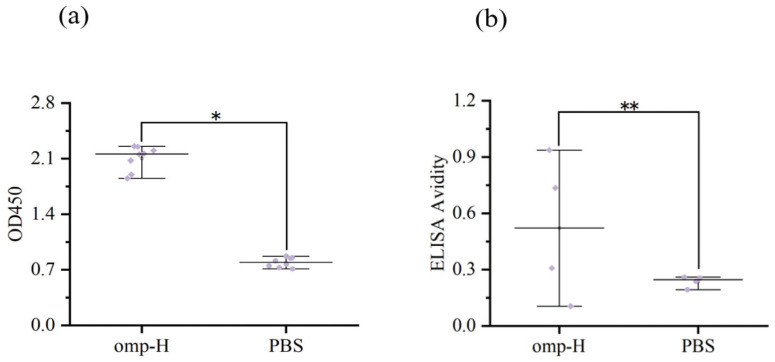
Serum IgM levels and avidity in *L. crocea* following Omp-H immunization. (**a**) Serum IgM levels determined by ELISA following Omp-H immunization. (**b**) Serum IgM avidity determined by ACE ELISA following Omp-H immunization (two-tailed *t*-test, * *p* < 0.05, ** *p* < 0.01).

Optimization of the ELISA protocol was carried out to identify optimal conditions for the concentration of coating antigen, the dilution of serum samples, and the concentration of the secondary antibody. A 1:1024 serum dilution was determined to be optimal for differentiating between positive and negative controls in *L. crocea* (Figure 1a). ELISA confirmed the presence of significantly elevated titers of IgM specific to Omp-H in the serum of immunized fish, relative to the PBS-injected controls (Figure 2a). The optical density followed a logarithmic decay with increasing concentrations of NH_4_SCN (R^2^ > 0.92). Serum samples from fish immunized with Omp-H exhibited minimal elution across the range of NH_4_SCN concentrations, indicating high-avidity antibodies (Figure 1b). The ELISA avidity index confirmed that antibodies induced by Omp-H immunization in *L. crocea* exhibited significantly higher avidity for their respective antigens compared to those in the control serum (Figure 2b). These ELISA results confirmed that Omp-H elicited both quantitative and qualitative enhancements in the specific IgM response, characterized by significantly elevated antibody titers and superior binding avidity compared to controls.

### 2.2. Serum IgM Levels and Avidity Measured by BLI

**Figure 3:**
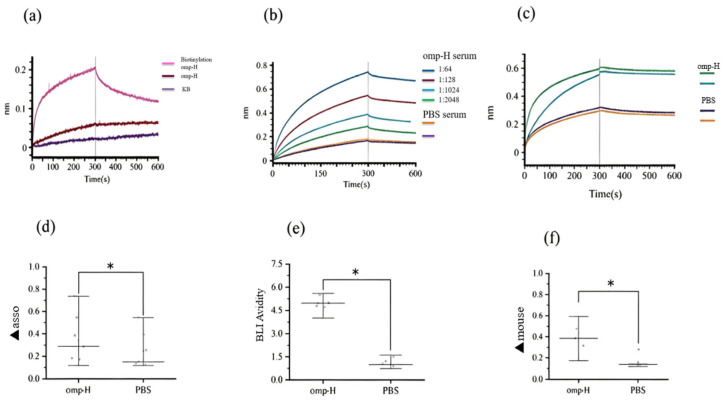
Results of serum IgM level and avidity measurements by BLI. (**a**) Non-specific binding of buffer KB and non-biotinylated proteins to the streptavidin biosensor. Association (0–300 s), dissociation (300–600 s). (**b**) Determination of the association (0–300 s) and dissociation (300–600 s) of *L. crocea* serum with outer membrane proteins on the streptavidin biosensor. (**c**) Detection of the association and dissociation of mouse anti-*L. crocea* IgM monoclonal antibody on streptavidin biosensors following the binding of *L. crocea* serum and outer membrane proteins. (**d**) Wavelength shift observed during association of serum IgM. (**e**) BLI avidity index, calculated as the ratio of association to dissociation (▲asso/▲disso) for each serum sample. (**f**) Wavelength shift following subsequent binding of mouse anti-IgM secondary antibody. Asterisks denote statistically significant differences between antigen-immunized and PBS-injected fish (two-tailed *t*-test, * *p* < 0.05).

At higher concentrations of immobilized protein, saturation curve profiles were observed for biotinylated Omp-H. The association (▲asso) and dissociation (▲disso) curves for the negative controls, involving running buffer (KB) or non-biotinylated proteins on the SA sensor surface, indicated an absence of non-specific binding (Figure 3a). A serum dilution of 1:128 was determined to be suitable for sample analysis, as determined by the association curve profiles and signal consistency (Figure 1a). To quantify serum IgM levels in *L. crocea*, the BLI ▲asso was measured (Figure 3b), and specific binding to the ligand was verified using a mouse anti-IgM monoclonal antibody (▲mouse). Applying this approach with immobilized Omp-H revealed that ▲asso values were substantially higher in the serum of immunized fish compared to the PBS control group (Figure 3d). The ▲mouse was also significantly higher in immunized groups compared to the PBS control, where ▲mouse values did not exceed 0.3 nm (Figure 3c,e). According to the BLI avidity index, the antibody avidity in *L. crocea* immunized with Omp-H was significantly higher than that in PBS-injected controls (Figure 3f). Although ▲asso primarily reflects antigen-specific IgM binding, it may theoretically include contributions from other specific-binding proteins, such as other antibody isotypes (e.g., IgT) or non-IgM serum components that interact with the immobilized antigen. Therefore, the ▲mouse signal obtained with the anti-*L. crocea* IgM monoclonal antibody serves as a critical control to confirm that the measured ▲asso indeed corresponds to IgM. In this study, the strong correlation between ▲asso and ▲mouse (R^2^ = 0.81) suggests that the contribution of non-IgM binding to ▲asso is minimal under our experimental conditions. Collectively, these BLI results validated the method’s capacity to detect antigen-specific IgM with minimal background interference, while successfully distinguishing immunized from control groups based on both antibody levels (▲asso) and binding avidity (avidity index).

### 2.3. Evaluating the Performance of ELISA and BLI in Fish Serum IgM Analysis

**Figure 4:**
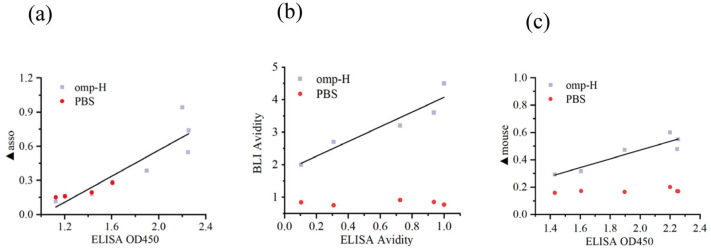
Comparative analysis of serum anti-Omp-H IgM levels, avidity, and secondary antibody binding in *L. crocea* using ELISA and BLI. (**a**) Comparison of specific anti-Omp-H serum IgM levels determined by ELISA and BLI methods, *p* < 0.05, R^2^ = 0.77. (**b**) Comparison of anti-Omp-H serum IgM avidity calculated by ACE ELISA and BLI methods, *p* < 0.05, R^2^ = 0.85. (**c**) Correlation between ELISA absorbance and BLI Omp-H serum IgM binding to mouse anti-*L. crocea* IgM monoclonal antibody, *p* < 0.05, R^2^ = 0.81.

**Figure 5:**
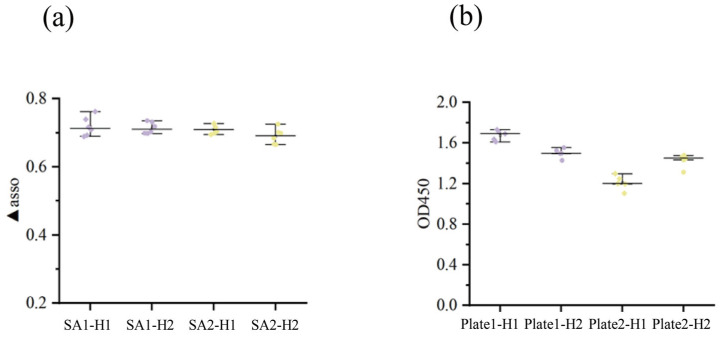
Inter-plate and inter-well variability analysis of BLI and ELISA measurements. (**a**) Inter-plate and inter-well differences observed in BLI measurements. (**b**) Inter-plate and inter-well differences observed in ELISA measurements.

Data obtained from the BLI assay were in excellent agreement with those from ELISA, validating the reliability of this novel approach for fish IgM analysis. Specifically, for Omp-H, the ▲asso and ▲mouse values correlated strongly with the corresponding ELISA absorbance readings, with R^2^ values of 0.77 and 0.81, respectively (Figure 4a,c). Moreover, a strong linear relationship was observed between the ELISA and BLI avidity indices, with R^2^ values exceeding 0.7 (Figure 4b). This finding suggests that the ▲asso/▲disso ratio serves as a dependable surrogate for conventional avidity measurements, capturing the functional avidity maturation of the antibody response. Regarding reproducibility, BLI showed excellent consistency across replicate plates, whereas ELISA suffered from significant inter-plate variation (Figure 5a,b). These findings validate the ▲asso/▲disso ratio as a dependable surrogate for ELISA-based avidity measurements, capturing functional avidity maturation with the added advantage of enhanced reproducibility and reduced inter-assay variability.

### 2.4. Specificity of the Mouse Anti-L. crocea IgM Monoclonal Antibody Against Different Fish Sera

**Figure 6:**
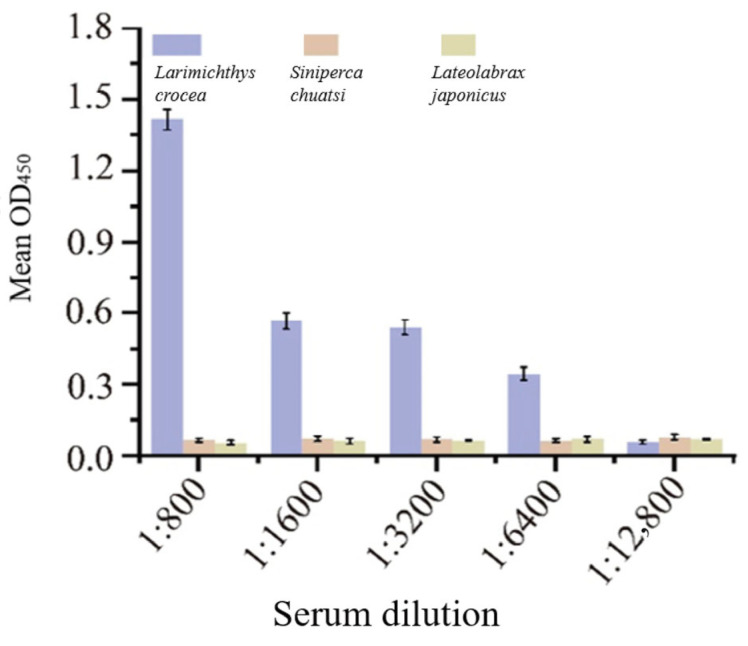
Specific detection results of mouse anti-large yellow croaker (*L. crocea*) IgM monoclonal antibody.

The specificity of the mouse anti-*L. crocea* IgM monoclonal antibody was evaluated by ELISA using sera from large yellow croaker (*L. crocea*), Chinese perch (*Siniperca chuatsi*), and Japanese seabass (*Lateolabrax japonicus*). As shown in Figure 6, the anti-IgM monoclonal antibody specifically recognized IgM molecules in the serum of large yellow croaker (*L. crocea*). Compared to the sera from Chinese perch (*Siniperca chuatsi*) and Japanese seabass (*Lateolabrax japonicus*), the experimental group exhibited a significantly higher absorbance at 450 nm, demonstrating clear specific binding. In the antibody dilution gradient experiment, a detectable effect was still observed when the antibody dilution ratio was 1:6400. These results confirm that the prepared monoclonal antibody exhibits good immunoreactivity and specificity, providing a reliable experimental basis for establishing an immunoassay for large yellow croaker (*L. crocea*) IgM.

### 2.5. Validation of the BLI Method Using Omp-W Protein and Its Application in Detecting Sera from Live Bacterial Infection

**Figure 7:**
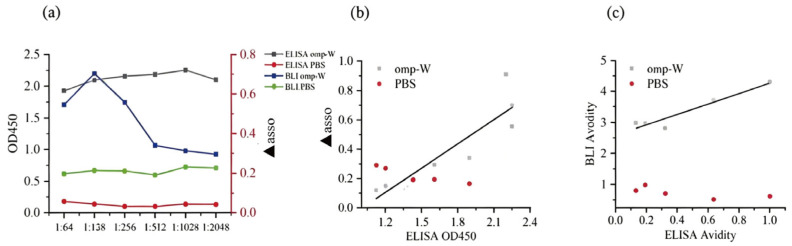
Correlation between ELISA and BLI for the detection of serum anti-Omp-W IgM levels and avidity following immunization. (**a**) Correlation between serum ELISA absorbance at 450 nm (left axis) and BLI wavelength shift (▲asso, right axis) following immunization with Omp-W. (**b**) Comparison of specific anti-Omp-W serum IgM levels determined by ELISA and BLI methods, *p* < 0.05, R^2^ = 0.77. (**c**) Comparison of anti-Omp-W serum IgM avidity calculated by ACE ELISA and BLI methods, *p* < 0.05, R^2^ = 0.89.

**Figure 8:**
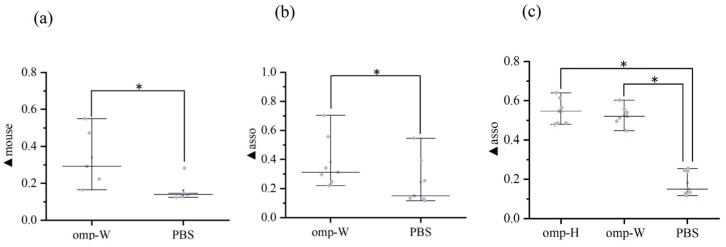
BLI wavelength shifts analysis of serum IgM binding and secondary antibody recognition following bacterial immunization in *L. crocea*. (**a**) Wavelength shift observed during association of serum IgM. (**b**) Wavelength shift following subsequent binding of mouse anti-IgM secondary antibody. (**c**) Wavelength shifts observed during the binding of serum IgM following *P. plecoglossicida* immunization. Asterisks denote statistically significant differences between antigen-immunized and PBS-injected fish (two-tailed *t*-test, * *p* < 0.05).

To validate the general applicability of the BLI method, *L. crocea* were immunized with another outer membrane protein of *P. plecoglossicida*, Omp-W, which was also used as an immobilized ligand for parallel detection. The results showed that the binding signal (▲asso) between sera from the Omp-W-immunized group and the Omp-W biosensor was significantly higher than that of the PBS control group (*p* < 0.05), and the kinetic profiles were similar to those observed with Omp-H detection (Figure 7a,b). A serum dilution of 1:128 was determined to be optimal for differentiating between positive and negative controls. The secondary binding signal of the monoclonal antibody (▲mouse) also showed significant differences, with ▲mouse values in the Omp-W immunized group being significantly higher than those in the PBS control group (Figure 8a). Meanwhile, comparison of the BLI and ELISA results revealed a strong positive linear correlation between the BLI binding signal (▲asso) for the Omp-W antigen and the corresponding ELISA absorbance values, with R^2^ values of 0.77, respectively (Figure 7b). The BLI avidity index for the Omp-W antigen also showed a positive correlation with the ELISA avidity index (R^2^ > 0.8) (Figure 7c). These results demonstrate that the BLI method can effectively detect specific antibodies induced by different outer membrane protein antigens, exhibiting good universality. These findings demonstrate that the BLI method maintains excellent performance characteristics, including optimal signal differentiation at 1:128 serum dilution, specific binding verification via ▲mouse, and strong cross-platform correlations when applied to different antigenic targets, establishing its versatility for fish immunology studies.

To further evaluate the applicability of the two outer membrane proteins as detection antigens, serum samples from live bacterial infection were analyzed. Following live *P. plecoglossicida* infection, the levels of anti-Omp-H and anti-Omp-W specific IgM antibodies in *L. crocea* serum exhibited changes. The ▲asso signals for both antigens were elevated and consistently higher than those of the PBS control group (Figure 8c). The dynamic trends of antibody responses detected by the two antigens were generally consistent, indicating that both Omp-H and Omp-W can serve as detection antigens for monitoring antibody responses following live bacterial infection. These results demonstrate that the BLI method based on Omp-H and Omp-W is effective for detecting antibody responses following *P. plecoglossicida* infection. The elevated ▲asso signals for both Omp-H and Omp-W in infected sera, coupled with their consistent dynamic trends, demonstrated that BLI effectively captures infection-induced antibody responses, validating the practical utility of both outer membrane proteins as diagnostic antigens [21].

## 3. Discussion

The present study describes a novel method for measuring specific IgM and its avidity for antigens in the serum of *L. crocea* following *P. plecoglossicida* infection using BLI. This BLI analysis yielded results comparable to those of ELISA. Both ELISA and BLI proved effective for quantifying the relative levels of IgM specific to the immunizing antigens in fish serum. The observation of significant differences between the Omp-H, Omp-W and PBS-injected groups clearly demonstrated the biological effects of the immunization. The strong positive correlation between the BLI data and ELISA results indicates that ▲asso is an acceptable parameter for quantifying serum IgM levels [22,23].

To verify the binding specificity to Omp-H and Omp-W on the SA biosensor, a mouse anti-*L. crocea* IgM secondary antibody was used. The ▲mouse results confirmed that the measured ▲asso was indicative of *L. crocea* IgM bound to the biosensor. Nevertheless, it is worth noting that in the absence of a specific secondary antibody, ▲asso could theoretically include contributions from other proteins that specifically bind the immobilized antigen, such as other antibody isotypes (e.g., IgT) or certain serum components. Therefore, IgM levels estimated solely from ▲asso might be overestimated in some cases. In the present study, the strong correlation between ▲asso and ▲mouse (R^2^ = 0.81) suggests that such non-IgM binding did not substantially affect our quantification. A major hurdle in direct antibody analysis from crude serum lies in the non-specific binding and background signals that compromise assay specificity [24]. In ELISA, blocking agents, including BSA, skim milk, or animal serum, are used in combination with extensive washing procedures to eliminate non-specific binding and remove residual reagents from the plate surface [25]. In BLI, blocking is not required, as negative control serum on blank SA sensors shows minimal background signal arising from serum proteins to streptavidin [26]. In this study, the mouse anti-*L. crocea* IgM-specific antibody was used solely to verify that the signal detected by BLI corresponded to the antibody response. Binding kinetics are reflected in the BLI avidity index, which is derived from the ratio of association to dissociation [19]. A higher ▲disso corresponds to lower overall IgM binding avidity when ▲asso values are similar between two serum samples [19]. However, this assumes that the shape of the association and dissociation curves is invariant between samples, with differences arising solely from specific epitope avidity [27]. In this experiment, when two samples exhibited slight inconsistencies in their association curves, the shape of their dissociation curves remained very similar. Notably, this avidity model has limitations. In samples with extremely low IgM concentrations, the correspondingly low ▲asso and ▲disso values may result in a misleadingly elevated avidity index. This potential artifact can be mitigated by carefully optimizing analyte titrations with appropriate positive and negative biological controls. Taken together, these findings demonstrate that BLI enables reliable relative quantification of IgM avidity in teleost serum.

The direct one-step dip-and-read BLI technology provided reliable data on relative antibody levels and avidity in this study. Furthermore, the BLI method was validated using the Omp-W protein and applied to the detection of sera from live bacterial infection. These results demonstrate that the BLI detection system is independent of specific antigen types, exhibiting good universality and scalability. The minimal reagent requirements of the BLI procedure yielded highly reproducible results with minimal intra- and inter-plate variability. It is important to note that the broader distribution of BLI ▲asso values (0.1–0.7 nm) compared to ELISA OD450 values (1.8–2.2) reflects BLI’s unamplified, linear detection preserving natural biological variance, rather than greater technical variability. As shown in Figure 5, the inter-plate coefficients of variation for BLI were consistently lower than those for ELISA, confirming its superior reproducibility. In practice, biological replicates alone were sufficient to effectively demonstrate the treatment effects [28]. This consistency facilitates data comparison across fish immunology projects, provided that the BLI methodology is standardized [21]. Absorbance-based detection in ELISA, achieved through biological amplification of antibody signals, is prone to interference from abiotic factors, including temperature variations, pH deviations, incubation timing errors, and suboptimal washing conditions, all of which can compromise data accuracy. However, the optimal serum dilutions differed between platforms (1:1024 for ELISA vs. 1:128 for BLI), reflecting their distinct detection principles: ELISA relies on enzymatic amplification, requiring higher dilutions to avoid signal saturation, while BLI directly measures unamplified binding, necessitating higher IgM concentrations (lower dilution) for detectable signals. This difference does not compromise cross-platform comparability, as evidenced by the strong correlations shown in Figure 4. When serum is limiting or antibody levels are low, ELISA may provide more efficient quantification [29]. From a cost-effectiveness standpoint, BLI provides immediate experimental data without the need for specific secondary detection antibodies.

In conclusion, BLI’s excellent universality and scalability are of great significance for serological detection following pathogen infection in fish for pathogenic strains with different serotypes or antigenic variations. A specific antibody detection method can be rapidly established simply by replacing the corresponding biotinylated antigen protein, without the need for re-optimization of the experimental protocol. This approach provides more reproducible data and offers substantial potential for high-throughput applications.

## 4. Materials and Methods

### 4.1. Larimichthys crocea (Large Yellow Croaker)

*Larimichthys crocea* (Large yellow croaker), (300–500 g) were obtained from the Fujian Fuding Research Center of the East China Sea Fisheries Research Institute, Chinese Academy of Fishery Sciences. The fish were acclimated and maintained in an experimental recirculating aquaculture system (RAS) at 27 °C. Water quality parameters, including temperature, salinity, dissolved oxygen, pH, ammonia, nitrite, and nitrate, were monitored regularly. Partial water exchanges were performed when any of these parameters deviated from the optimal ranges.

### 4.2. Preparation of Experimental Vaccines, Fish Immunization, and Serum Harvest

The gene sequences encoding the outer membrane proteins Omp-H of *P. plecoglossicida* were obtained [30]. Oligonucleotide primers specific to the target genes were designed for subsequent PCR amplification: 5′-CCGGAATTCATGAAAACCTTCAACACAC-3′ and 5′-CCCAAGCTTTCAGAACTTGTAGTTCGC-3′. The amplified fragments were then cloned into the pET30a vector to generate the recombinant plasmids pET30a-Omp-H for subsequent protein expression in *E. coli*. The size of the whole bacterial protein after IPTG induction is approximately 25.0 kDa [31]. For immunization, the recombinant Omp-H proteins were used as antigens in *L. crocea* [32]. The antigens were emulsified with adjuvant at a 3:7 ratio (*v*/*v*, antigen: Freund’s adjuvant), yielding a final concentration of 0.1 mg/mL for each antigen.

Feeding was suspended for 24 h prior to immunization. The experimental fish were anesthetized with eugenol at a density of 25–30 mg/L. On day 0, fish in the immunization groups (n = 50 per antigen) received an intraperitoneal injection of the immunogen (200 μL/fish), while fish in the control group (n = 50) were injected with an identical volume of phosphate-buffered saline (PBS). On day 21, the same fish in the immunization groups received a booster injection of the respective immunogen (200 μL/fish) to stimulate a secondary antibody response. At day 28 post-immunization, blood samples were obtained from each fish via the caudal vein [33]. After overnight storage at 4 °C to enable clotting, serum was isolated via centrifugation (3800× *g*, 5 min) and maintained at −80 °C pending further analysis.

### 4.3. Fish Serum IgM Was Measured by ELISA

An indirect ELISA was employed to quantify antigen-specific antibodies in fish serum [34]. The procedure described below was implemented under the established ELISA conditions for fish serum detection [35,36]: The Omp-H proteins were diluted to 10 μg/mL in coating buffer, applied to high-binding 96-well ELISA plates at 100 μL per well, and then incubated at 37 °C for 2 h. To block nonspecific binding sites, the samples were incubated overnight at 4 °C with 200 μL/well of 5% skim milk. After three consecutive washes with TBST (Tris-buffered saline with 0.05% Tween 20, pH 7.5; 100 μL/well), diluted serum samples (at dilutions of 1:64, 1:128, 1:256, 1:512, 1:1024, and 1:2048) were added in triplicate (100 μL/well) and incubated at 37 °C for 1 h [37]. Three washes with TBST were performed, after which the mouse anti-*L. crocea* IgM monoclonal antibody (diluted to 1 μg/mL in antibody diluent; 100 μL/well) was applied and incubated at 37 °C for 1 h. The washing step was repeated, followed by addition of HRP-conjugated goat anti-mouse IgG (1:3000 dilution in antibody diluent; 100 μL/well) and incubation at 37 °C for 1 h. Color development was then carried out, and absorbance at 450 nm was recorded using a 96-well spectrophotometer [38].

### 4.4. Fish Serum IgM Avidity Was Determined via ACE ELISA

To assess the avidity of antigen-specific antibodies in serum, an ACE ELISA was performed based on a modified version of the protocol described above. Five independent sets of triplicate wells on the ELISA plate were designated for each fish serum sample, which was diluted 1:1024 prior to addition. Following serum incubation and subsequent washes, ammonium thiocyanate (NH_4_SCN) at concentrations of 0, 0.05, 0.1, 0.3, and 0.5 g/mL was added to the respective sets of triplicate wells (50 μL/well) and incubated at room temperature for 15 min [39]. After three consecutive rinses with TBST, the plates were processed following the detection steps outlined in Section 4.3 without modification. For each serum sample, the OD values were plotted against the NH_4_SCN concentration, and a logarithmic decay model was fitted to the data using Origin 8.5 (OriginLab Corporation, Northampton, Massachusetts, United States), defined as the NH_4_SCN concentration required to halve the initial OD value (at 0 g/mL NH_4_SCN). The ELISA avidity index served as a measure of antibody binding strength [19].

### 4.5. Fish Serum IgM Levels and Avidity Were Determined via BLI

**Figure 9:**
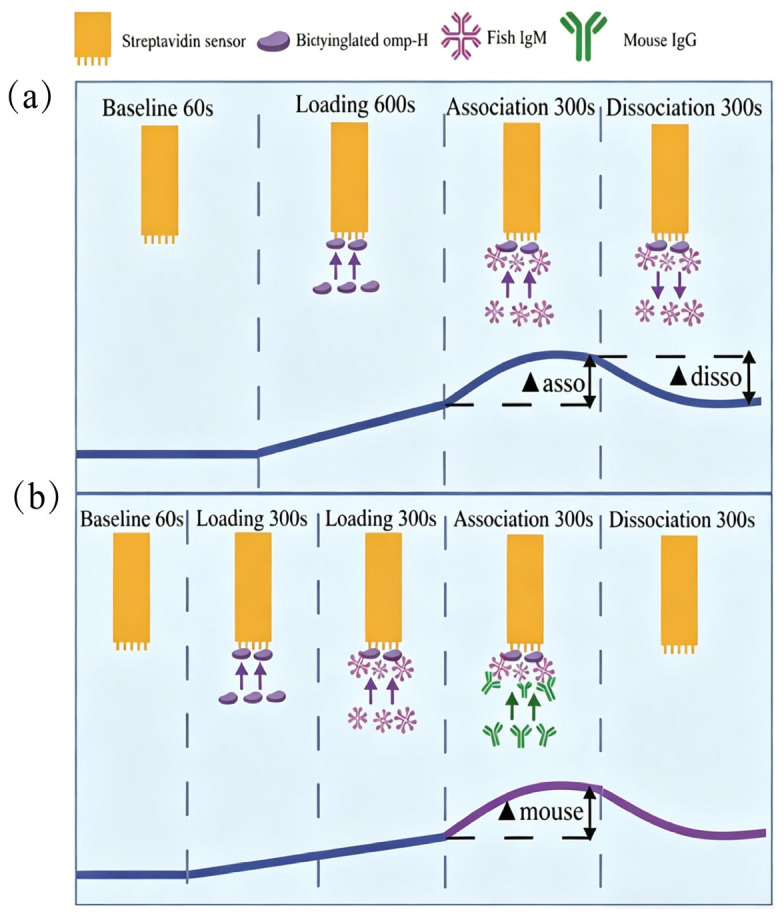
Schematic illustration of the Bio-Layer Interferometry (BLI) workflow using the Octet^®^ R2 system [22]. (**a**) The dark blue line represents the baseline signal, generated when the streptavidin biosensor is immersed in a well containing the running buffer (TBST; Tris-buffered saline with 0.05% Tween 20). Subsequently, biotinylated Omp-H proteins are loaded onto the streptavidin biosensor. The loaded biosensor is then immersed in sample wells containing diluted *L. crocea* serum (analyte) for the association step, during which specific IgM in the serum binds to the Omp-H-immobilized sensors. The sensor is then moved to a well containing TBST for the dissociation step, where weakly bound IgM dissociates from the antigen. Optical variations on the sensor are recorded in real time through interferometric detection. The resulting wavelength shifts during the association (▲asso) and dissociation (▲disso) phases are then employed to assess relative IgM concentration and avidity in serum. (**b**) The purple line represents an additional BLI step, where a mouse anti-*L. crocea* IgM monoclonal antibody (diluted 1:50) is added to validate the specific interaction with target IgM (▲mouse). This secondary association step is unnecessary for routine quantification of serum IgM.

The real-time binding profiles of serum IgM from *L. crocea* were measured using the Octet^®^ R2 system (Figure 9). All assays were performed in standard Greiner black 96-well microtiter plates, with each well containing a final volume of 200 μL. According to the manufacturer’s specifications, Omp-H was biotinylated using a biotinylation kit at a 20:1 molar ratio (biotin:protein). The biotinylated proteins were immobilized onto Streptavidin (SA) biosensors. Fish serum (analyte) was diluted in 1× Kinetics Buffer (KB) at dilutions of 1:64, 1:128, 1:256, 1:512, 1:1024, and 1:2048 [40]. The secondary mouse anti-*L. crocea* IgM monoclonal antibody was diluted to 1 μg/mL for use. Before commencing the experiment, hydration of the streptavidin (SA) biosensors was performed in 1× Kinetics Buffer (KB) for 10 min. The baseline, association, and dissociation steps were all performed in 1× KB.

All data were processed with the Octet^®^ R2 Data Analysis software. The raw sensor data were first corrected by subtracting the mean reference sensor value. To quantify the wavelength shift, the data were aligned to the Y-axis at the initiation of the association step. The wavelength shift was obtained by averaging 10 data points, corresponding to a 2-s interval. The BLI avidity index was calculated using the following formula:

BLI Avidity = (▲asso/▲disso)(1)

### 4.6. Specificity Detection of Mouse Anti-L. crocea IgM Monoclonal Antibody by ELISA

To evaluate the specificity of the mouse anti-*L. crocea* IgM monoclonal antibody used in this study, an indirect ELISA was performed with serum samples from three different fish species: large yellow croaker (*L. crocea*), Chinese perch (*Siniperca chuatsi*), and Japanese seabass (*Lateolabrax japonicus*). All serum samples were diluted at ratios of 1:800, 1:1600, 1:3200, 1:6400, and 1:12,800, respectively, subsequently coated onto 96-well plates, and then incubated at 37 °C for 1 h. After blocking with 5% skim milk, the samples were incubated with mouse anti-crocodile IgM monoclonal antibody (1 μg/mL) at 36 °C for 1 h, followed by incubation with HRP-conjugated goat anti-mouse IgG (1:3000) for 1 h. Color development was then carried out, and absorbance at 450 nm was recorded using a 96-well spectrophotometer.

### 4.7. Validation and Application of the BLI Method Using P. plecoglossicida Outer Membrane Protein Omp-W

To validate the universality and specificity of the biolayer interferometry (BLI) method, parallel validation experiments were conducted using another outer membrane protein, Omp-W, from *P. plecoglossicida*. Specific oligonucleotide primers were designed for the target gene: 5′-CCGGAATTCATGAGAAGACTGTTTG-3′ and 5′-CCCAAGCTTTCAGAAGCGATAGCCGAGGGTG-3′, for subsequent PCR amplification. The amplified fragments were then cloned into the pET30a vector to generate the recombinant plasmids pET30a-Omp-W for subsequent protein expression in *E. coli*. The size of the whole bacterial protein after IPTG induction is approximately 28.5 kDa [31]. The Omp-W protein was biotinylated and loaded onto streptavidin (SA) biosensors following the procedure described in Section 2.5. BLI detection was performed using the same *L. crocea* serum samples as those used in the Omp-H immunization experiment (including the Omp-W immunized group and the PBS control group), at dilutions of 1:64, 1:128, 1:256, 1:512, 1:1024, and 1:2048. The feasibility of the BLI method for detecting different antigen-specific antibodies was evaluated by comparing the binding kinetics parameters (including wavelength shifts during association and dissociation) of the Omp-W biosensor with sera from different immunization groups [28].

To further validate the applicability of this method in an actual infection model, serum samples were collected from *L. crocea* following live *P. plecoglossicida* infection. Using the optimized BLI protocol described above, specific IgM antibodies in the post-infection sera were detected with both Omp-H and Omp-W protein biosensors. The sensitivity and specificity of these two outer membrane proteins in detecting antibody responses after live bacterial infection were evaluated by analyzing the binding kinetic characteristics of the serum samples. Additionally, the BLI results were compared with those obtained by the ELISA method described in Section 2.3 to verify the reliability of BLI technology in monitoring post-infection antibody dynamics. All assay conditions, including temperature, baseline time, association time, and dissociation time, were kept consistent with those described in Section 2.5 to ensure data comparability [16].

## 5. Conclusions

In this study, a biolayer interferometry (BLI)-based method was successfully established for detecting specific IgM antibodies in *L. crocea* serum and systematically compared with traditional ELISA. Following optimization, optimal serum dilutions were determined as 1:128 for BLI and 1:1024 for ELISA, with no significant non-specific binding observed. For antibody quantification, both BLI association signal and monoclonal antibody secondary binding signal effectively distinguished immunized from control groups, showing strong positive linear correlations with ELISA absorbance values. In avidity assessment, the BLI avidity index correlated strongly with the ELISA elution-based index, confirming that BLI simultaneously provides information on antibody quantity and quality. Validation using two distinct outer membrane proteins (Omp-H and Omp-W) demonstrated good antigen universality. Application to infected serum samples revealed antibody response dynamics consistent with ELISA results, validating reliability for practical infection monitoring. Compared with traditional ELISA, BLI technology offers faster detection speed, simpler operation, elimination of enzyme-labeled secondary antibody steps, and, most importantly, exceptionally high inter-assay reproducibility, overcoming the inherent inter-plate variation limitation of ELISA. Although ▲asso alone may overestimate IgM levels in the absence of isotype-specific validation, the inclusion of ▲mouse measurements in this study ensured specificity. This method provides a rapid, reliable, and standardized detection platform for serological surveillance, vaccine efficacy evaluation, and antibody dynamics studies in farmed fish.

## Data Availability

The original contributions presented in this study are included in the article. Further inquiries can be directed to the corresponding authors.

## Abbreviations

The following abbreviations are used in this manuscript:

BLI	Biolayer Interferometry
ELISA	Enzyme-linked immunosorbent assay
IgM	Immunoglobulin M
RAS	Recirculating aquaculture system
